# Potassium-Induced Phenomena and Their Effects on the Intrinsic Reactivity of Biomass-Derived Char during Steam Gasification

**DOI:** 10.1021/acsomega.3c02234

**Published:** 2023-08-01

**Authors:** Saiman Ding, Efthymios Kantarelis, Klas Engvall

**Affiliations:** Department of Chemical Engineering, KTH Royal Institute of Technology, Stockholm SE-10044, Sweden

## Abstract

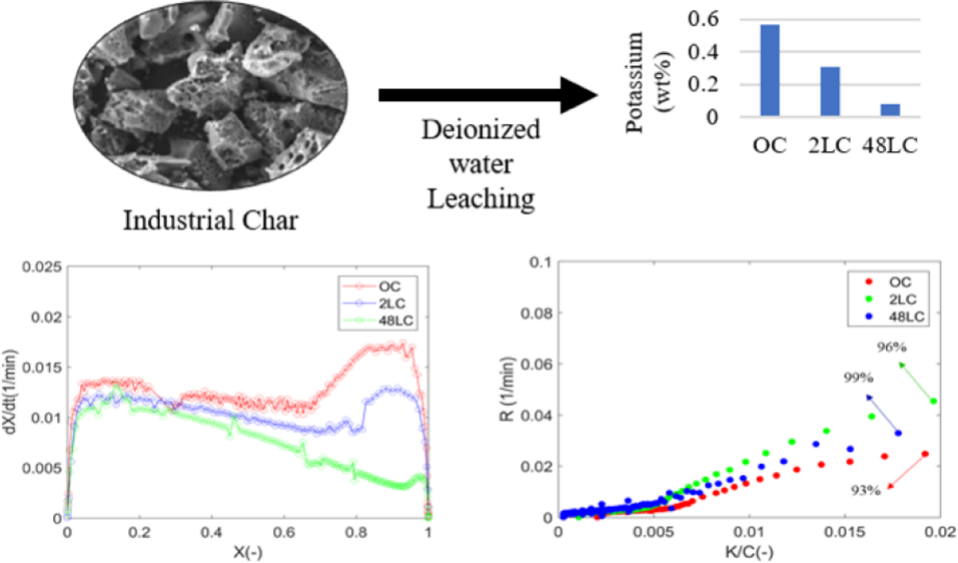

The mineral content of biomass plays an important role in the gasification rate of biomass-derived char. The understanding and quantification of mineral-related phenomena are thus of importance when considering gasification reactor design. In the present work, the potassium-induced catalytic phenomena during gasification of biomass-derived char have been studied. Char samples with similar structure and different intrinsic potassium content were gasified in a steam atmosphere at a temperature range of 700–800 °C. It was found that for all the samples, irrespective of the temperature and the initial potassium content, there is a critical K/C ratio (5 × 10^–3^), whereafter the catalytic phenomena prevail. The instantaneous conversion rate of the char is positively correlated with the potassium content and the progressively increasing conversion. The application of the modified random pore model was able to capture the later stages of conversion by the introduction of two additional parameters (*c* and *p*). It was found that these constants are not just fitting parameters but that there is an underlying physical significance with *c* being directly related to the intrinsic potassium content while being temperature independent and with *p* being temperature dependent.

## 1 Introduction

Gasification is a thermochemical process enabling the conversion of lower-grade carbonaceous solid materials, such as biomass, to producer gas/syngas that in turn can be used for power generation and/or fuels, as well as chemical production.^1^ Char conversion (i.e., oxidation of solid carbon to gaseous compounds) is considered the rate-determining step for such conversion,^2^ and hence, an understanding of the governing mechanisms and kinetics of char gasification is important for gasification reactor design.^3^

The literature abounds with studies using different biomass chars gasified at different conditions and exhibiting different reactivities. Those differences are related to (a) the parent biomass (composition and structure) and (b) the char formation conditions (devolatilization conditions, temperature history of particles, etc.). The latter results in different morphology and textural properties, as well as variable speciation and quantity of minerals in the resulting char.^4−24^

During gasification, char undergoes structural changes, such as generation of new pores, enlargement and coalescence of existing ones, particle fragmentation,^19,20,25^ etc., all of which influence the specific surface area, porosity, and pore size distribution^21,23,26^ and affect the accessibility of the oxidizing agents to the inner particle during the char conversion.^23,27^ When it comes to minerals, alkali and alkaline earth metals (AAEMs) are of importance.^4−7,14,24,28^ For biomass-based feedstocks, among the inherent AAEM species, potassium (K) is of greatest interest^4,5^ followed by calcium (Ca), which is typically found as a carbonate or an oxide.^29−33^ The importance of potassium lies in the fact that it enhances the gasification rate by forming active potassium-oxygen complexes.^34−38^ The role of Ca is however unclear with literature reporting (a) enhancement of pore structure development for coal chars during gasification^39,40^ and (b) inhibition of potassium deactivation^39,41,42^ and hence gasification rate promotion, especially in the presence of steam,^41^ plausibly due to the formation of Ca-K active compounds.^39,42^ Nevertheless, the catalytic activity of Ca is manifested at early stages of char conversion (*X* < 0.4), whereas K enhances the reaction rate at the later stages,^43−45^ with K being indisputably more active toward char gasification than Ca.^14,46^ Other elements influencing the catalytic char gasification reactivity are iron (Fe), sodium (Na), and magnesium (Mg), but their role in the overall conversion is limited because of their low abundance and reduced (or even inhibiting) activity compared to potassium.^47,48^

Because of the complex nature of overlapping phenomena, it is often difficult to distinguish between different contributions in the observed reactivity. Consequently, the use of well-defined systems minimizing the number of parameters while analyzing biochar gasification even though imperative is still challenging. To decouple the effects of the minerals from inherent structure of biochars, many studies have used leaching and impregnation techniques to modify the mineral content of the char.^44,49−51^ Nevertheless, impregnation as a technique can result in inaccurate localization and/or improper speciation of the impregnated minerals as opposed to the native ones.^44,52^

In a previous study,^3^ an important ash-related rate enhancement at the later stages of the char conversion was observed. The present work extends the understanding and provides insights by quantifying those phenomena (and particularly the ones related to potassium) on industrial biomass derived char (char derived from entrained flow gasification; more details in can be found elsewhere^3^) during steam gasification.

Contrary to prior research where the role of potassium in char gasification is explored, this study uniquely focuses on rapidly pyrolyzed biomass in industrial gasifier and quantifies the specific phenomena associated with potassium-induced activity. Decoupling between structural and catalytic phenomena is attempted by analyzing char samples with similar morphology and varying intrinsic potassium contents. The study establishes correlations between observed gasification rates and the availability and reactivity of char relative to potassium. Additionally, the research employs kinetic modeling to accurately quantify the potassium-induced activity enhancement under conditions relevant to industrial applications. The quantification of the potassium-induced activity enhancement is carried out by kinetic modeling at industrially relevant conditions. Different kinetic models are evaluated, and a physical interpretation of obtained results is given. To the best of the authors’ knowledge, this issue has not been studied previously for industrial biomass char.

## 2 Methods

### 2.1 Raw Material and Sample Preparation

The raw material used was unreacted biochar collected from an industrial-scale entrained flow gasifier (Meva Energy AB, Sweden), gasifying <0.1 mm pine biomass particles in a temperature range of 900–1150 °C Prior to use, the sample was ground to a particle size of 45–120 μm. The resultant char is designated as original char (OC).

Biochars with different amount of mineral content, so-called leached char (LC) samples, were prepared using deionized water as a leaching agent. The detailed leaching procedure can be found elsewhere.^3^ The leached samples are designated as 2LC and 48LC and refer to 2 and 48 h of treatment, respectively.

### 2.2 Characterization of the Chars

The chars were analyzed by means of proximate and ultimate analyses, whereas the mineral matter of the chars was analyzed by inductively coupled plasma sector field mass spectrometry (ICP-SFMS). The specific surface area was determined by N_2_ adsorption (Micromeritics ASAP 2000) using the BET (Brunauer–Emmett–Teller) method. The surface of chars was examined by scanning electron microscopy–energy-dispersive X-ray spectroscopy (SEM–EDX). A Si (Li) detector and the Oxford INCA Energy software were used to determine the surface element concentrations.

The chemical composition and specific surface area of the original char and LC samples are reported in Table 1.

**Table 1:** Properties of Original Char and Leached char^3^

	OC	2LC	48LC
Proximate analysis (wt %)			
moisture	0.600	0.240	0.000
volatile matter (db)^a^	4.530	3.980	3.510
ash (db)^a^	6.900	5.300	3.400
fixed carbon (db)^b^	88.570	90.720	93.090
Ultimate analysis (wt % db^c^)			
C	85.770	87.280	87.920
H	1.120	0.950	0.880
N	0.370	0.360	0.360
S	0.090	0.070	0.060
O^b^	5.760	6.040	7.380
Mineral content (wt % db)			
Na	0.076	0.045	0.017
K	0.569	0.306	0.077
Mg	0.266	0.260	0.235
Ca	1.120	1.020	0.932
Fe	0.072	0.062	0.060
Al	0.034	0.034	0.033
Mn	0.154	0.155	0.153
P	0.068	0.070	0.069
Si	0.145	0.192	0.085
specific surface area (m^2^/g)	330 ± 1.87	340 ± 2.16	410 ± 2.56

a Determined at 550 °C.

b Calculated by the difference.

c db: dry basis.

The ICP-SFMS analysis shows that K (0.57 wt %), Mg (0.26 wt %), and Ca (1.12 wt %) are the major mineral elements in the OC, whereas Na (0.076 wt %) and Fe (0.072 wt %) are present in lower concentrations. The water leaching is mostly effective in removing alkali metals (K and Na) and more specifically K. The treatment resulted in 46.2 and 86.5% reduction of K after 2 and 48 h of leaching, respectively, whereas in the case of Na, a reduction of 34.4 and 77.1% after 2 and 48 h of treatment, respectively, is observed. Removal of other elements such as Mg, Fe, and Ca is considerably milder with a decrease of 2.2 and 11.6% for Mg, 14.4 and 16.8% for Fe, 8.9 and 16.7% for Ca after 2 and 48 h of treatment, respectively.

### 2.3 Thermogravimetric Analysis

Char gasification experiments were carried out under isothermal conditions using a NETZCSH ST490 F3 thermogravimetric analyzer (TGA). The sample was heated in a N_2_ atmosphere (300 mL/min) at a heating rate of 10 °C/min up to a temperature of 900 °C and treated isothermally for 3 h to remove any residual volatile matter. Then, the samples were cooled to the desired temperature (700, 750, and 800 °C) at which it was maintained for 5 min; after signal stabilization, steam (7.6 mol %) was injected. It is expected that the conversion is the chemical reaction control regime at the investigated reaction conditions.^3,53^ In each experiment, 30 ± 2 mg of char powder was used. For each of the runs, duplicates were made showing satisfactory repeatability (±2%).

To compare the gasification rates at different conversions (*X*), the conversion rate (*dX*/*dt*) is normalized with respect to the unconverted fraction (1 – *X*) (expressed as *R*, instantaneous conversion rate). The char conversion (*X*), conversion rate (*dX*/*dt*), and instantaneous conversion rate (*R*) are defined using eqs 1–3, respectively:
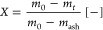
1
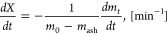
2
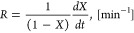
3

where *m*_0_ represents the initial mass of the char, *m_t_* is the instantaneous mass of the char at time *t*, and *m*_ash_ is the remaining mass of ash.

### 2.4 Kinetic Modeling

The kinetics of gasification is generally described as a combination of the effects of operating conditions and char structure, where eq 4 represents the kinetics of a reaction:^54,55^
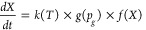
4*k*(*T*) is the apparent reaction rate constant, and the *g*(*p_g_*) function indicates the dependence of the reactivity on the partial pressure of the gasifying agent. *X* is the conversion, and *f(X)* describes the structure change that is dependent on the conversion. In the present study, the partial pressure (*p_g_*) of the gasifying agent was kept constant.

The reaction rate constant is only temperature dependent and can therefore be defined by the Arrhenius equation (eq 5).
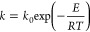
5*k*_0_ is pre-exponential factor, *E* is the activation energy (J/mol), *R* is the universal gas constant (J/mol/K), and *T* is the reaction temperature (K).

Three models were used to describe the steam gasification rate of the char samples, namely, the first-order pseudo-homogeneous model (HM), shrinking core model (SCM), and random pore model (RPM).

In the HM,^56^ the first-order reaction rate is proportional to the conversion. The model assumes that the steam reacts with the char at active sites, which are uniformly distributed throughout the particle. The structure changes during the reaction are not taken into consideration. The expression for reactivity according to HM is shown in eq 6.
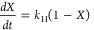
6

Szekely and Evans^57^ proposed a different model-SCM, assuming that a particle is of uniform nonporous structure and that the reaction takes place on the external surface. If the reaction is under chemical reaction control and the shape of the grain is spherical, the overall reaction rate is
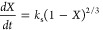
7

A model developed by Bhatia and Perlmutter^58^ (RPM) assumed that reactions happen both on the external surface and in the pores. The pores coalesce and simultaneously generate new ones as the carbon is consumed. The RPM expression is given below:^58^
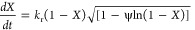
8

where ψ is known as a structure parameter related to the pore structure of the nonreacted sample, and it can be determined by the experimental maximum conversion values (0 ≤ *X_max_* < 0.393) according to^57,59^
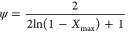
9

A modified model was developed based on RPM to include the catalytic effect of the minerals.^5,49^ According to this model, two empirical constants are introduced:
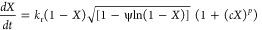
10*c* and *p* are dimensionless parameters used to describe the observed increase in the reaction rate due to the catalytic activity of the mineral content.^5^ Throughout the paper, this model modification is named as modified random pore model (MRPM).

Equations 6, 7, and 8 are linearized, resulting in eqs 11, 12, and 13, allowing the determination of the reaction rate constants at different temperatures from the slopes of the linear expression.
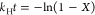
11
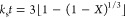
12
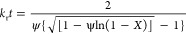
13

It should be noted that ψ is dependent on the initial structural properties of chars.^58^ It can be derived from the maximum conversion rate as given in eq 9.

The coefficient of determination, *R*^2^, is used as an indication of goodness of fit of different models compared with experimental data.
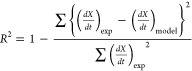
14

The nonlinear least-squares method in MATLAB was used to fit the experimental reaction rate results to estimate the kinetic parameters and the *R*^2^.

## 3 Results and Discussion

### 3.1 Char Reactivity in Steam Atmosphere

**Figure 1:**
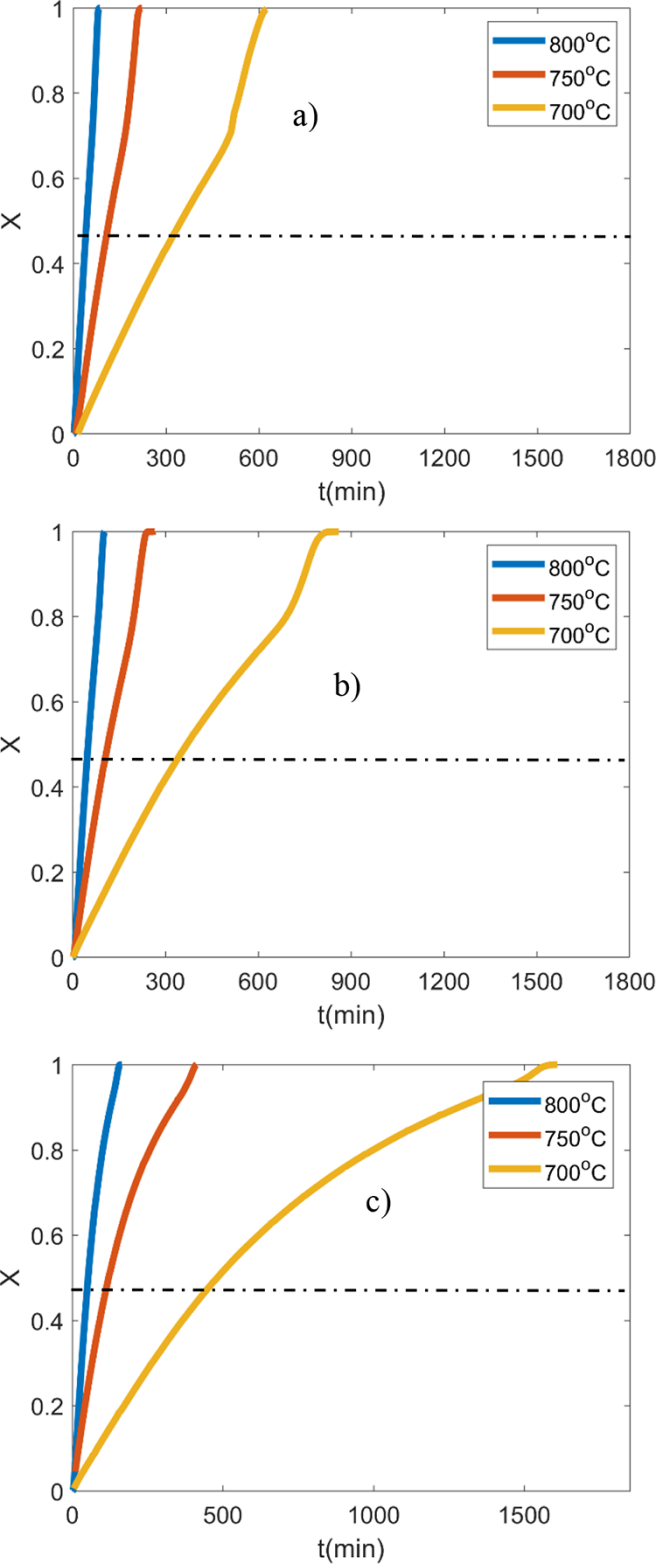
Gasification time versus conversion of three different chars: (a) OC, (b) 2LC, and (c) 48LC.

Figure 1 presents the conversion of the samples at different temperatures for the three char samples. As expected, the temperature is positively correlated with the conversion rate for all the chars.

At the lower temperature (i.e., 700 °C), there is a negative correlation between the time for achieving 50% conversion and the mineral content of the chars as it is extended by 5 and 38% for the 2LC and 48LC samples, respectively, compared to the OC. The same is observed at 750 °C where 1.7 and 5% longer reaction time is needed for the 2LC and 48LC chars, respectively, compared to OC. Similarly, at 800 °C, 14 and 23% more time to achieve 50% conversion for 2LC and 48LC is needed. This observation is more pronounced for the complete conversion of chars with the 2LC and 48LC samples requiring 38 and 140% more time compared to the OC at 700 °C. At 750 °C, the corresponding time for the 2LC and 48LC is extended by 11 and 86%, respectively, whereas at 800 °C, the reaction time reaches 123 and 190% of the OC, respectively. This indicates that the presence of minerals affects the conversion rate toward the later stages of the char conversion.

**Figure 2:**
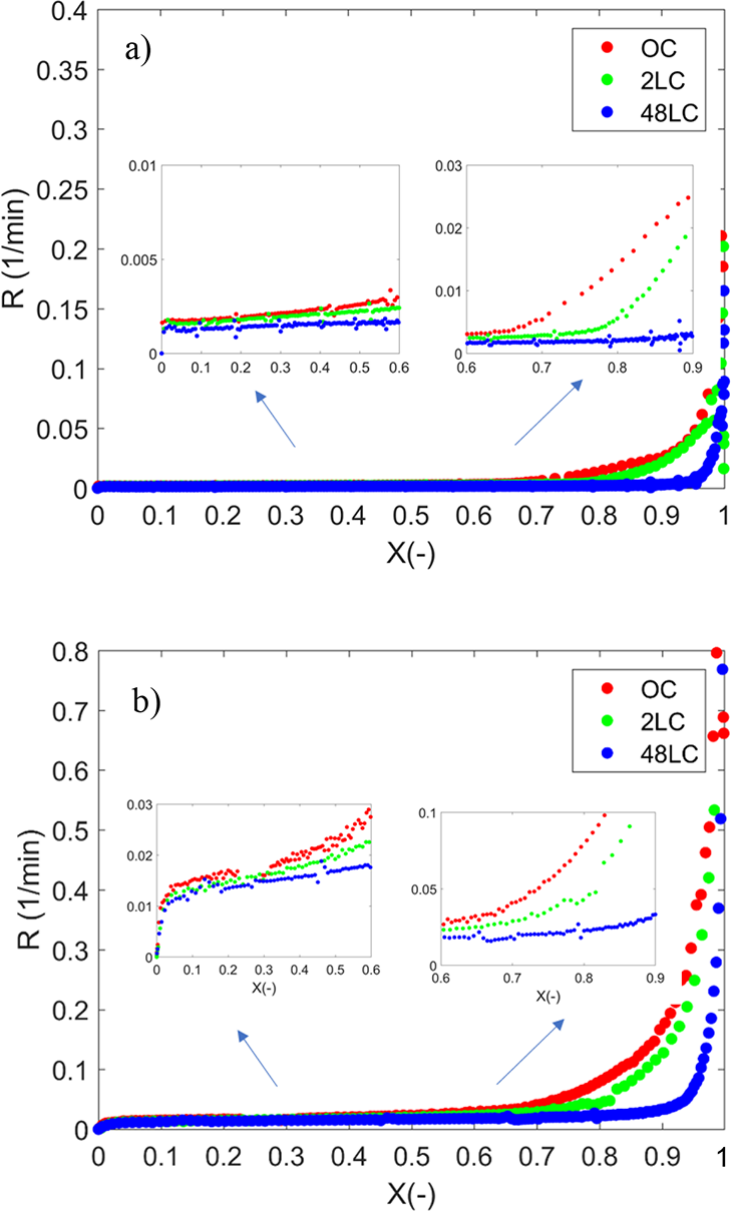
Instantaneous char gasification rates of all samples at (a) 700 °C and (b) 800 °C as a function of the conversion.

Figure 2a depicts the instantaneous conversion rate, *R*, of all samples as a function of the degree of conversion at 700 °C (the corresponding graphs for 750 °C can be found in the Supporting Information (Figure S1). As shown, the *R* of all the samples is essentially the same until a certain degree of conversion, followed by a rapid increase with progressing conversion. The onset varies with the degree of deashing and is observed at conversions of 67, 78, and 92% for the OC, 2LC, and 48LC, respectively (Figure 2 right inset) at 700 °C and is directly correlated to the difference in potassium content of the chars.^5,28,60,61^ At higher temperatures, the onset is shifted to a lower degree of conversion, as illustrated for OC and 48LC at 800 °C in Figure 2 b. This shift is rather small in the case of 48LC, indicating that potassium is the main reason for the larger shift observed for OC and 2LC (see also Figure S2 in the Supporting Information for a better display of the shift due to temperature).

**Figure 3:**
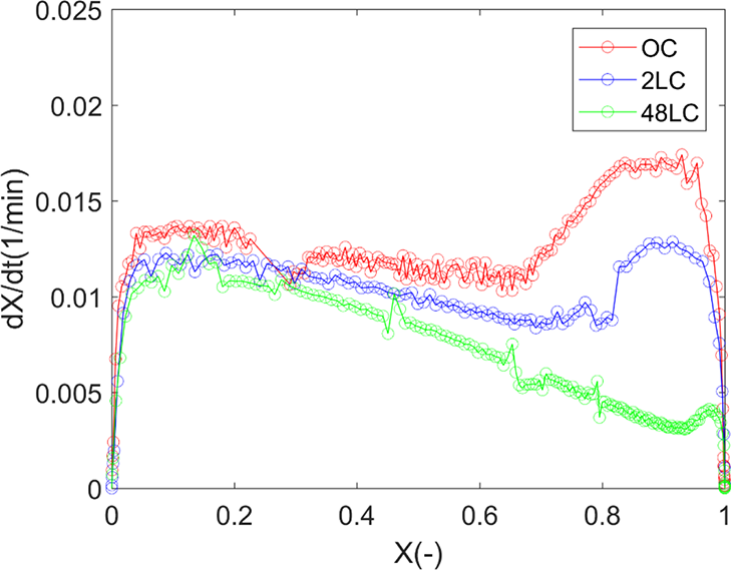
Experimental reactivity rate vs *X* for OC, 2LC, and 48LC at 800 °C.

At 700 °C, the observed difference in *R* for the three chars is small with a subsequently faster increase in *R* with higher potassium contents below *X* < 0.6, as displayed in Figure 2 a. At 800 °C, the instantaneous rate is sharply increased at the initial stage of conversion, as shown in Figure 2 b (left insert), also observed at 750 °C (see Supporting Information) but to a lower degree, whereas it was not found for 700 °C. In view of the experimental reactivity rate versus conversion, this results in a conversion progression exhibiting two peaks, as displayed in Figure 3, a pattern also observed in other studies.^62,63^

The peak at a low conversion is plausibly attributed neither to catalytic activity by potassium^45^ nor to the initial specific surface area. In the latter case, the observed reaction reactivity *R* for each char shows the opposite trend with the highest for OC and the lowest for 48LC compared to the changes in specific surface areas (Table 1). Effects of Ca on the biomass char reactivity is another potential explanation, as reported by other studies,^43,44,64^ enhancing the reactivity at lower conversion by reacting with other ash compounds and thus preventing deactivation of the potassium.^43,64,65^ Most of these studies are however performed under a CO_2_ atmosphere. Other studies on coal char steam gasification,^39,40,66^ have shown the importance of CaO dispersion^67^ and form of Ca species^39,40^ in promoting the development of a porous char structure. As shown in Figure 3, the rapid initial increase followed by a plateau in conversion below 0.2 can be attributed to the substitution of the inert gas with the gasifying agent.^68^ This can be related to the opening of the structure and increased diffusivity enabled by steam. Moreover, the presence of steam, along with the dispersion of CaO,^67^ facilitates the formation of a porous structure, which offers a larger surface area and interconnected pathways. This structure enables enhanced diffusion of reactants and thus increase in the overall reaction rate.

After the initial peak at *X* < 0.2, the reaction rates for the chars diverge up to the rapid increase relates to the catalytic effect of potassium.^63^ The observed variation in reaction rates between 0.2 and 0.7 of conversion for the water-leached chars can be attributed to differences in the content of water-soluble calcium species, as indicated in Table 1. These differences may lead to variations in the development of the porous structure, with higher Ca content promoting larger surface area.^67^ Consequently, the reactivity of the OC sample is the highest, followed by the 2LC sample, with the 48LC sample showing the lowest reactivity.

The role of the ash minerals Fe and Mg on the gasification reactivity result as observed in Figure 3 is less likely. The catalytic effect of Mg is generally rather low at 800 °C, as shown in a study by Sadhwani et al.,^69^ who observed a very low catalytic effect with a Mg-loaded (1 wt %) pine char during CO_2_ gasification at this temperature. In the present study, the Mg content is only 0.26 wt %, and thus, the effect on the gasification reactivity can be considered negligible. A similar reasoning can be applied for Fe; although Fe is known to enhance the direct interaction between carbon and H_2_O,^70^ effects are generally observed for much higher Fe contents^70−72^ compared to the char samples used in the present study.

### 3.2 Effect of Potassium on Reactivity

**Figure 4:**
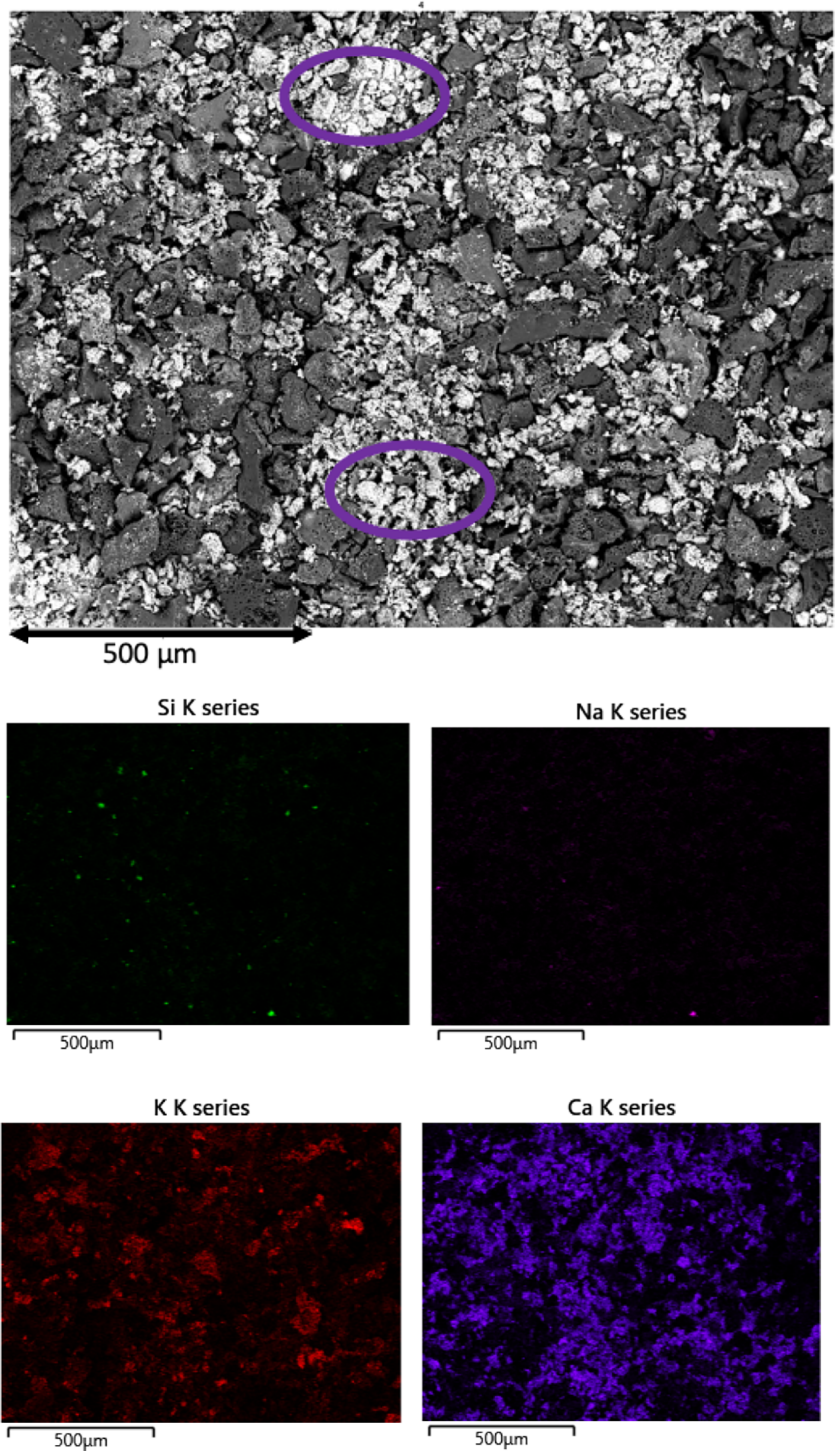
SEM image and EDS mapping of 75% converted OC at 750 °C.

**Figure 5:**
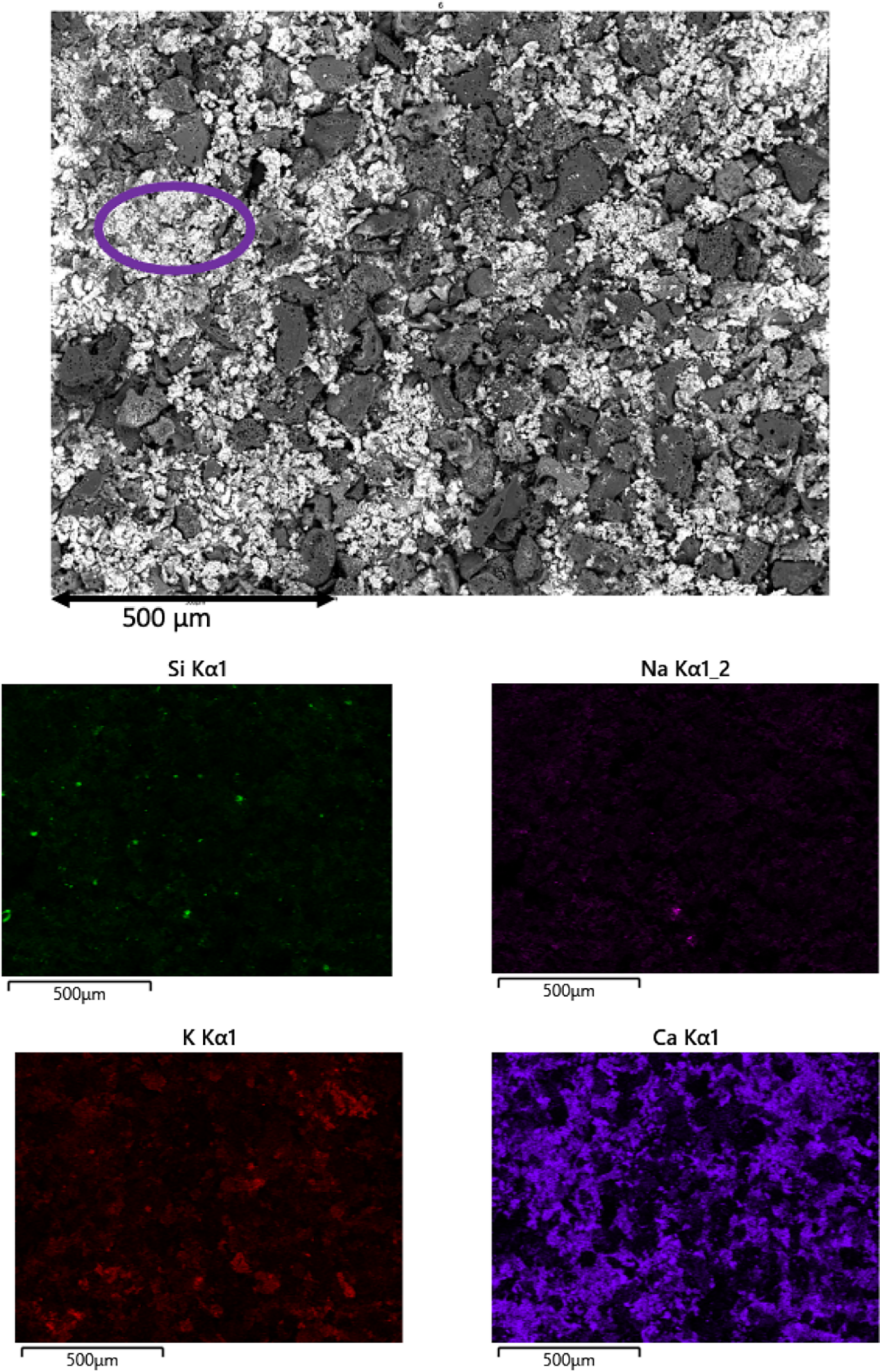
SEM image and EDS mapping of 80% converted 2LC at 750 °C.

To understand the effect of potassium on the increased reactivity at the later stages of the conversion, SEM mapping of OC and 2LC at later stages of conversion, *X* ≫ 75% and *X* ≫ 80%, respectively, was performed. Examples of SEM and EDS mapping results for Si, K, Na, and Ca are shown in Figures 4 and 5, respectively. Additional elements are found in Figures S6 and S7 in the Supporting Information. From the EDS mapping results, it is evident that the inorganic elements present at the sample surfaces primarily are Ca, O, K, Mg, Mn, and Fe at this late stage of conversion, as shown in Table 2. Patches of high content of Ca are clearly visible in the SEM image, as highlighted in purple circle for both conversions. It is also clearly visible in the EDS mapping of Ca in Figures 4 and 5. Si and Na are to a lesser extent present at the surface compared to the major elements.

**Table 2:** EDS Mapping Elemental Composition (wt %) as Obtained for OC and 2LC at *X* ≈ 75% and *X* ≈ 80%, Respectively

samples	Ca	O	K	Mg	Mn	Fe	P	Si	Na	Al	S
OC	40.3	29.2	10.8	6.6	5.5	3.1	1.9	0.9	0.7	0.6	0.5
2LC	39.1	30.8	8.1	6.5	6.0	4.3	2.5	0.9	0.8	0.7	0.4

The EDS elemental mapping results of the char surfaces, shown in Figures 4 and 5, indicate that the formation of alkali silicates or aluminates is negligible, as only a small number of areas of Si or Al coexist with K on the surface. Therefore, the amount of potassium silicates and/or aluminates inhibiting the catalytic activity of potassium^14,47,73−75^ or acting as a diffusion barrier for the oxidizer^76^ can be regarded as insignificant. We can thus conclude that essentially all potassium available at the surface is catalytically active.

To further address the potassium surface availability and its effect on the char conversion, we evaluate our results in terms of the atomic K/C ratio.^47^ The use of the K/C ratio also presumes that no K is volatilized from the sample during the char conversion. In support of this, our recent study^77^ confirmed that no detectable release of K occurs during industrial char conversion of up to around 90% at temperatures above 800 °C. In view of this and the temperature history of the samples (gasification at a temperature up to 1150 °C and treatment at 900 °C prior to TGA), it can therefore be considered that no/minimal K is emitted up to a conversion of 90% in the present study.

**Figure 6:**
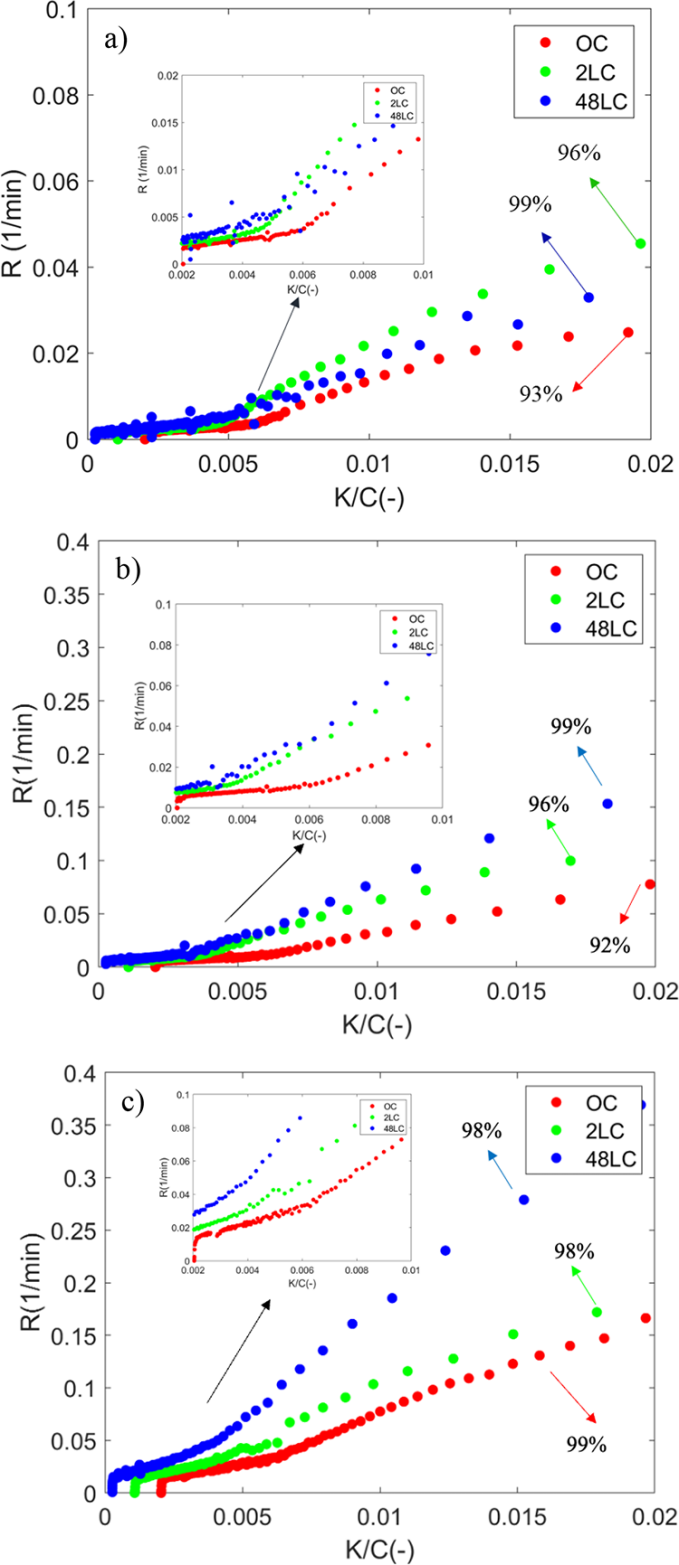
*R* versus the atomic K/C ratios from 0 to 0.02 for (a) 700 °C, (b) 750 °C, and (c) 800 °C (the percentages 92 to 99% inside the figures refer to conversion).

**Figure 7:**
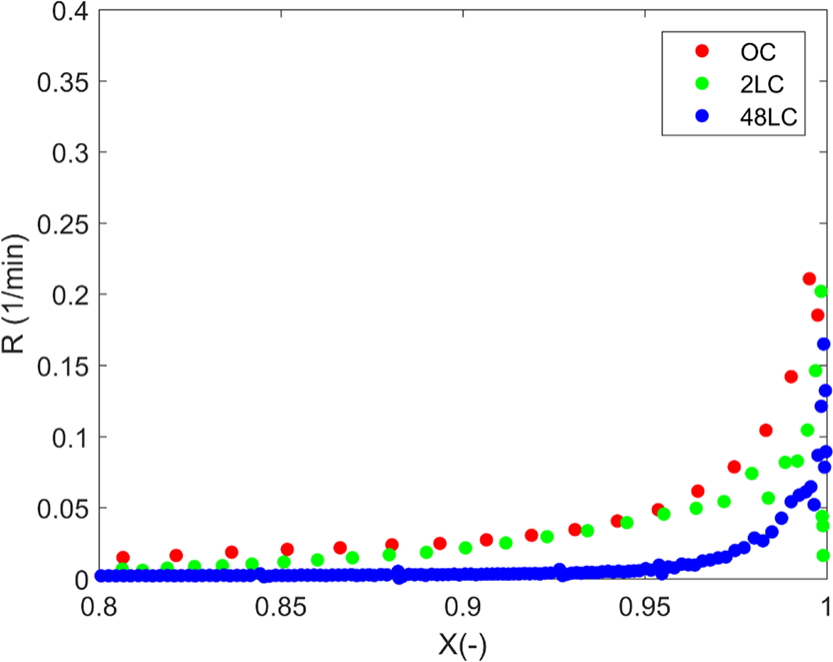
*R* of all samples at 700 °C as a function of conversion from 0.8 to 1.

Figure 6 shows the instantaneous conversion rate, *R*, as a function of K/C for the different char samples for all three temperatures investigated. The instantaneous reaction rates essentially increase slowly (relative change < 25%) for K/C ratios lower than approximately 0.005 for all samples. This part corresponds to a conversion lower than 70% (see also Figure 2). It is therefore suggested that the early-stage conversion is not governed by catalytic reactions, as supported by other studies.^78^ After this point, there is a sizeable monotonic increase of the instantaneous reaction rate for all temperatures. This indicates that there is a critical value of K/C ratio (active site availability) where the potassium plays an important catalytic role for the conversion of the char. At 700 °C (Figure 6a), the *R* follows a monotonic increase until complete conversion (*X* > 90% and K/C > 0.02) (Figures 6 and 7) for the 2LC and 48LC samples. In the case of the OC sample, a decrease in *R* is observed in the end toward complete conversion. The scenarios for 750 and 800 °C in Figure 6b,c, respectively, can be similarly described as for 700 °C.

In the present study, an inflection point of K where *R* starts to decrease, as identified for the high-ash containing char and potassium-impregnated coal char gasification,^47,63,79,80^ was not observed. Additionally, the determined increase of the instantaneous reaction rate as shown in Figure 6 is not proportional, as reported by Karlström et al.,^47^ who observed a proportionality between the instantaneous conversion rate and the K/C ratio in the conversion range of 0 to 80% during CO_2_ char gasification of K-rich agricultural biomass. In another study by Mims and Pabst,^80^ investigating the catalytic effect of impregnated potassium on the rate of coal char-CO_2_ gasification, three distinct reaction rate zones were observed. Also, in this case, a decrease in instantaneous conversion rate was observed at a later stage of the conversion (third zone).

There are considerable differences in ash composition comparing agricultural and woody char. Specifically, agricultural chars contain a much higher content of silicon, described as a catalyst deactivator,^14,73,75^ due to the strong thermodynamic affinity for potassium, and physically, a molten layer around the fuel particles might limit oxidizer access. Additionally, the abundant potassium content in agricultural chars leads to a higher ratio of K_2_O to total network formers (K_2_O, CaO, MgO), thereby facilitating the formation of potassium-rich silicates with lower melting temperatures.^81^ This may play an important role in case of the observed differences between the industrial woody char in the present and K and Si-rich agricultural chars. For char samples with impregnated K, the additional K to the char surface may block some of the pores and restrict gas access and thus the reaction sites, which may lead to a decrease in the rate.^80^ In conclusion, the result indicates that this value is sample specific (ash-K loading) and dependent of the temperature history of the char.

### 3.3 Kinetics of Char Gasification

To further validate the experimental results of the steam gasification activity, different kinetic models were employed and evaluated. There are several studies on char gasification kinetics using biochar employing commonly the volumetric reaction (VRM), the shrinking core (SCM), or the random pore (RPM) models.^49,56,82,83^ These models do not account for the effects of the inorganic content in the char.^5,51,83^ Different semi-empirical models,^5,45,51,84−86^ with the extended or the modified random pore model (MRPM) as one of the most widely used,^5,45,85^ have been proposed to address this issue. Studies of unreacted industrial char prepared to contain different intrinsic mineral contents, but with a similar morphology, applying these models are previously not reported.

**Figure 8:**
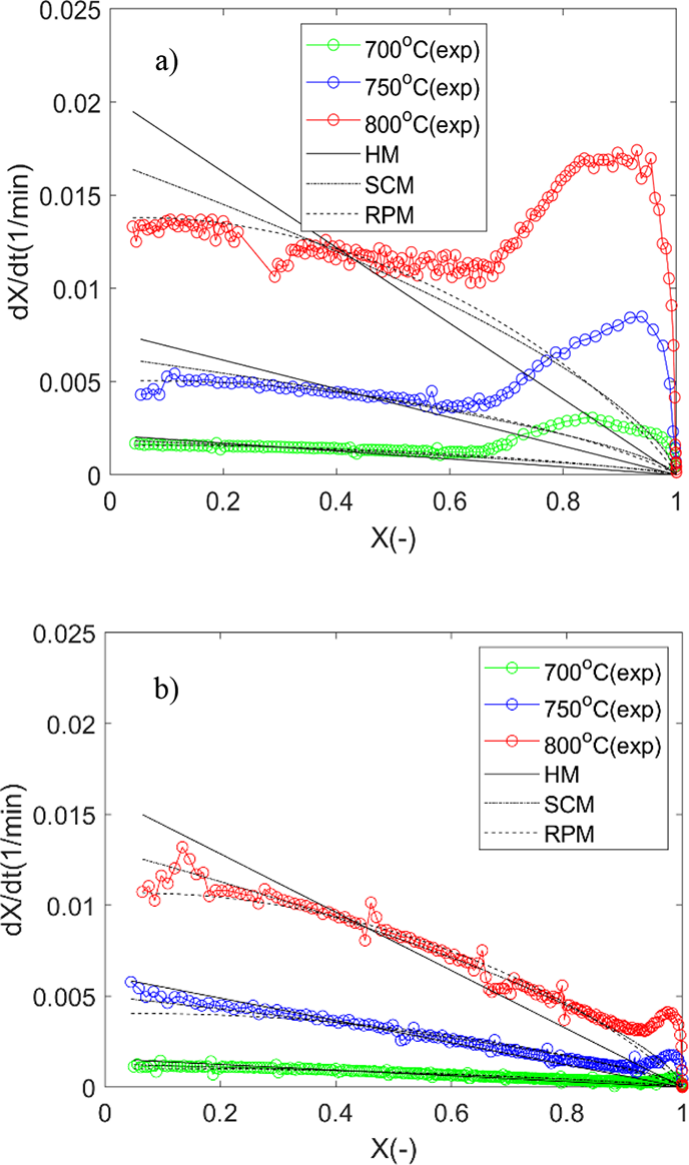
Comparison of the simulated and experimental data for gasification of (a) OC and (b) 48LC.

Table 3 lists the calculated activation energies and pre-exponential factors for the different models (HM, SCM, RPM) in their linearized forms (eqs 11–13) for all samples. Supplementary Information Figures S3 and S4 show the Arrhenius plot of three different models and the rate constant, respectively. The activation energy of three different chars for all the models is in the range of 191.65 ± 7.15 kJ/mol (confidence interval 99%), with similar values being reported in the literature under a chemical reaction control regime.^11,82,87,88^ The activation energy, calculated for all the models, is negatively correlated with the potassium content of the char. The difference in surface area of all the chars is small (<10%), leading to a slightly different maximum reaction rate at the early stage of conversion (Figure 3), but the effect on the ψ parameter can nevertheless be considered negligible. As shown in Table 3, the ψ parameter of the RPM model for the samples OC, 2LC, and 48LC equals to 2.28, 2.22, and 2.17, respectively, and was determined at 6, 5, and 4% conversion, which correspond to the maximum reaction rate (Figure 8).

**Table 3:** Kinetic Parameters of Char Steam Gasification for OC, LC, and 48LC Estimated by HM, SCM, and RPM

	HM		SCM		RPM		
samples	*E* (kJ/mol)	*k*_0_ (min^–1^)	*E* (kJ/mol)	*k*_0_ (min^–1^)	*E* (kJ/mol)	*k*_0_ (min^–1^)	ψ (−)
OC	184.70	1.87 × 10^7^	185.3	1.69 × 10^7^	186.1	1.43 × 10^7^	2.28
2LC	185.24	1.79 × 10^7^	186.2	1.68 × 10^7^	186.9	1.52 × 10^7^	2.22
48LC	201.58	9.9 × 10^7^	202.5	9.48 × 10^7^	203.1	8.39 × 10^7^	2.17

As shown in Figure 8, the RPM model describes the gasification process satisfactorily up to a varied degree of conversion depending on the K content and the temperature, followed by the SCM model. The HM model fails completely, especially for the higher temperatures 750 and 800 °C. For the conversions 70% for OC and 80 and 90% for 2LC (Figure S5) and 48LC, respectively, none of the models can describe the observed behavior. In case of the RPM, the predictive ability of the model is in direct relation to its potassium content for each gasification temperature. The effect of K becomes more pronounced as the temperature increases, displayed as a shift in the initiation of catalytic activity at a lower degree of conversion. The value for the structure parameter ψ is over 2 for all cases in Table 3, implying that a maximum of reaction rate versus conversion should be expected at conversions less than 0.4,^58^ as also observed in the present study.

**Figure 9:**
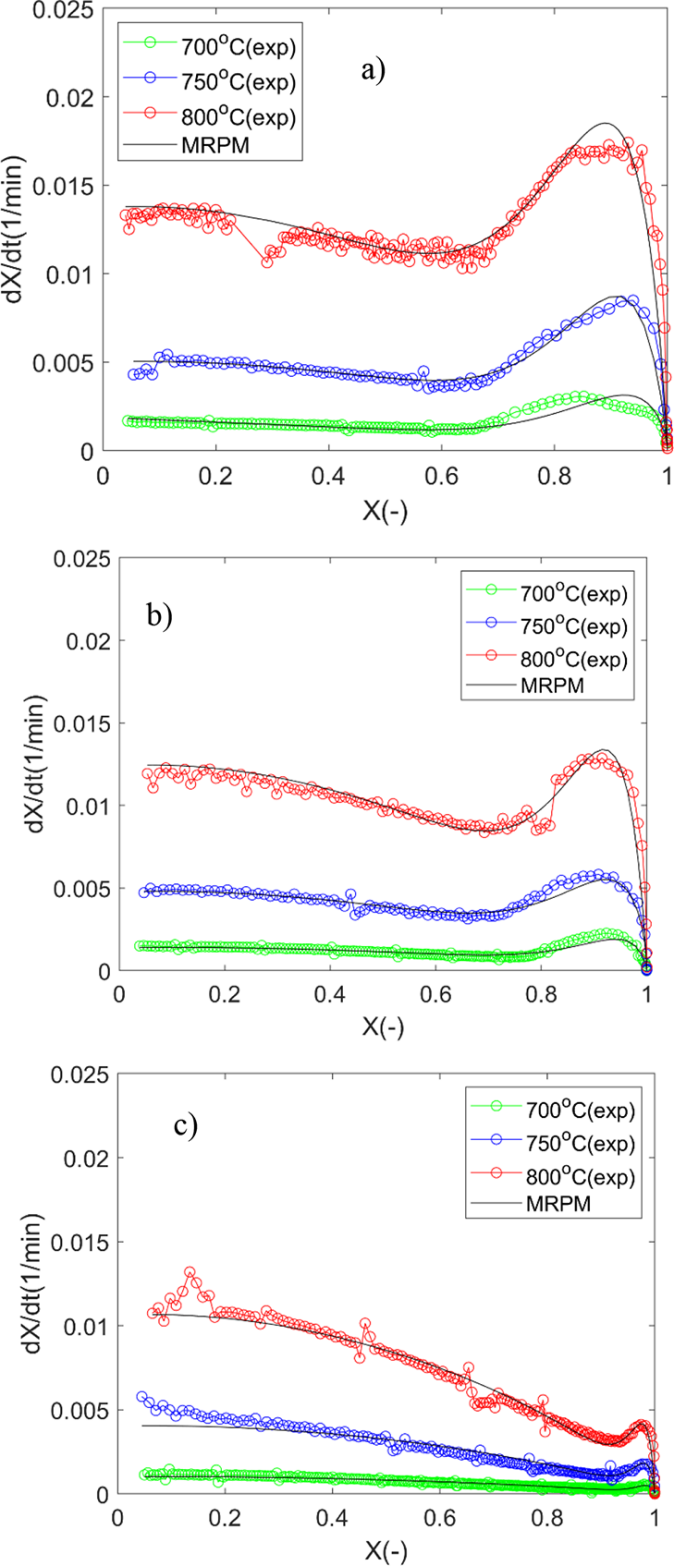
Experimental reactivity and the MRPM fitting as a function of conversion for (a) OC, (b) 2LC, and (c) 48LC.

To further model the experimental results considering effects of ash catalytic behavior during the steam gasification of the retrieved industrial char, a modified random pore model (eq 10) was used. The parameters *c* and *p* are related to the inorganic content^5^ and should enable a description of the later stages of conversion for all samples at the three different temperatures. From Figure 9, it is evident that the modified RPM is applicable to describe the entire conversion range, including the first peak potentially associated with Ca and its effect on pore development and the second peak ascribed to catalytic effects of potassium, for all cases.

The estimated kinetic parameters are listed in Table S1. The prediction of conversion rate using the MRPM is superior to all the other models (Figure 9). As shown, the *c* fitting parameter is constant (relative deviation < 3%) for each of the samples at the investigated temperature range (700–800 °C).

**Figure 10:**
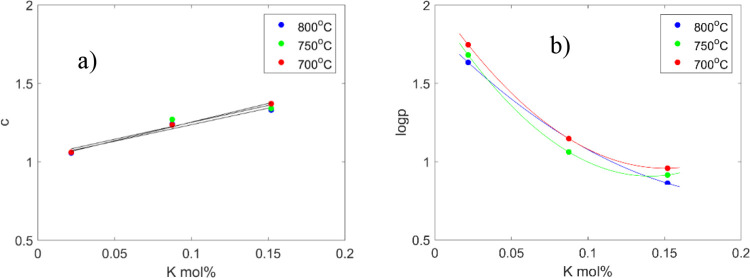
Relationships between potassium concentrations and the empirical constants (a) *c* and (b) *p* in the MRPM (trend lines as a guide for the eye).

The relationship between the K concentration and the two different empirical parameters *c* and *p* is shown in Figure 10, displaying a linear increase in *c* with increasing K concentration, whereas log*p* exponentially decreases for all temperatures.

In case of parameter *c*, Zhang et al.^5,45^ reported on similar results, investigating steam gasification of a series of chars from different biomasses at 850 °C and coal and carbon at 900 °C. In both these studies, a single temperature was used in the char gasification experiments. In the present study, we found that the parameter *c* is independent of the temperature used, as disclosed by the well collected points at each K concentration in Figure 10 a. The *p* parameter (Figure 10b) is also strongly dependent on the potassium content but also shows a small negative correlation with temperature. Nevertheless, the exponential decrease of log*p* vs K concentration differs from the results reported by Zhang et al.,^5,45^ with a linear decrease observed.

## 4 Conclusions

Investigation of the steam gasification of industrial wood char, with different intrinsic contents of potassium but with similar morphology, was carried out at a temperature range of 700–800 °C to understand the effects of the mineral and particularly potassium on the steam gasification rate. Experimental results were evaluated in terms of instantaneous char gasification rates as related to the potassium and carbon.

The instantaneous reaction rate, *R*, rapidly increases for all the samples tested at conversions higher than 0.6, with the onset being directly related to the potassium content as well as the temperature. The increase has been found to relate with K/C ratios, indicating that there is a critical value (common to all chars) above which a monotonic increase of the reaction rate is observed. On the basis of the critical K availability on the carbon surface, it can be said that at lower potassium contents, the char conversion is mainly controlled by the structural characteristics of the char (i.e., surface area) and plausibly by less active minerals, such as Ca. Given the monotonic increase in the reaction rate above the critical K/C concentration, it can be suggested that, contrary to high-ash chars, the surface is never fully saturated with potassium, which can also be related to the high degree of fixation (limited mobility) of K species due to the particle temperature history.

The MRPM can describe the entire conversion range, including the first peak potentially associated with Ca and its effect on pore development and the second peak ascribed to catalytic effects of potassium, for all cases. It has been revealed that the fitting constants have physical significance with the *c* parameter being directly related only to the potassium content, whereas for the second parameter *p*, a clear dependence on potassium content is observed with a simultaneous dependence on temperature.

## Supporting Information Available

Instantaneous char gasification rates and experimental reactivity; linearized plot of HM, SCM, and RPM models for chars; EDS mapping results for chars; relationship between K/C ratio and instantaneous gasification rate of chars; and kinetic parameters and regression coefficients estimated by the MRPM (PDF)

## Nomenclature

*A*: pre-exponential factor
*dX/dt*: gasification reactivity
*E*: activation energy, kJ kg^–1^ K^–1^
*f*(*X*): mechanism function
*k*: reaction rate constant
*k*_0_: pre-exponential factor, bar^–1^ s^–1^, bar^–1^
*p*_g_: gasification agent partial pressure
*R*: universal gas constant, 8.314 J mol^–1^ K^–1^
*R*_s_: gasification reactivity index
*R*^2^: correlation coefficient
*T*: reaction temperature, K
*t*: reaction time, s
*X*: char conversion rate
Ψ: structural parameter

## Abbreviations

OC: original char
2LC: 2 h leached char
48LC: 48 h leached char
HM: homogeneous model
SCM: shrinking core model
RPM: random pore model
MRPM: modified random pore model
