# Supplementary material for: Potassium-Induced Phenomena and Their Effects on the Intrinsic Reactivity of Biomass-Derived Char during Steam Gasification
Source: ACS Omega. 2023 Aug 1;8(32):29131–42. doi: 10.1021/acsomega.3c02234 (PMC10433354; doi:10.1021/acsomega.3c02234)
Supplement: Supplementary file 1 — ao3c02234_si_001.pdf [file ao3c02234_si_001.pdf]

Supporting Information

Potassium-induced phenomena and their effects on the intrinsic reactivity of biomass-derived char during steam gasification

Saiman Ding, Efthymios Kantarelis, Klas Engvall*

* To whom correspondence should be addressed kengval@kth.se (K. Engvall)

KTH Royal Institute of Technology, Department of Chemical Engineering, Stockholm, SE-10044 Sweden.

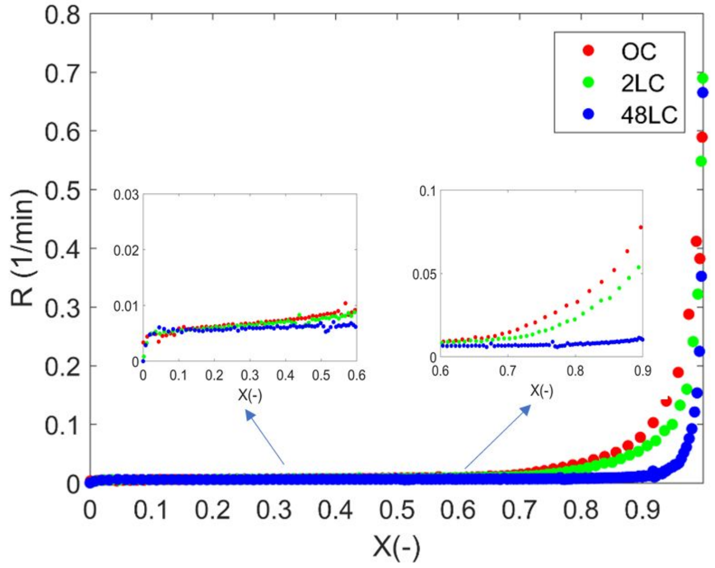

Figure S1. Instantaneous char gasification rates of all samples at 750 °C

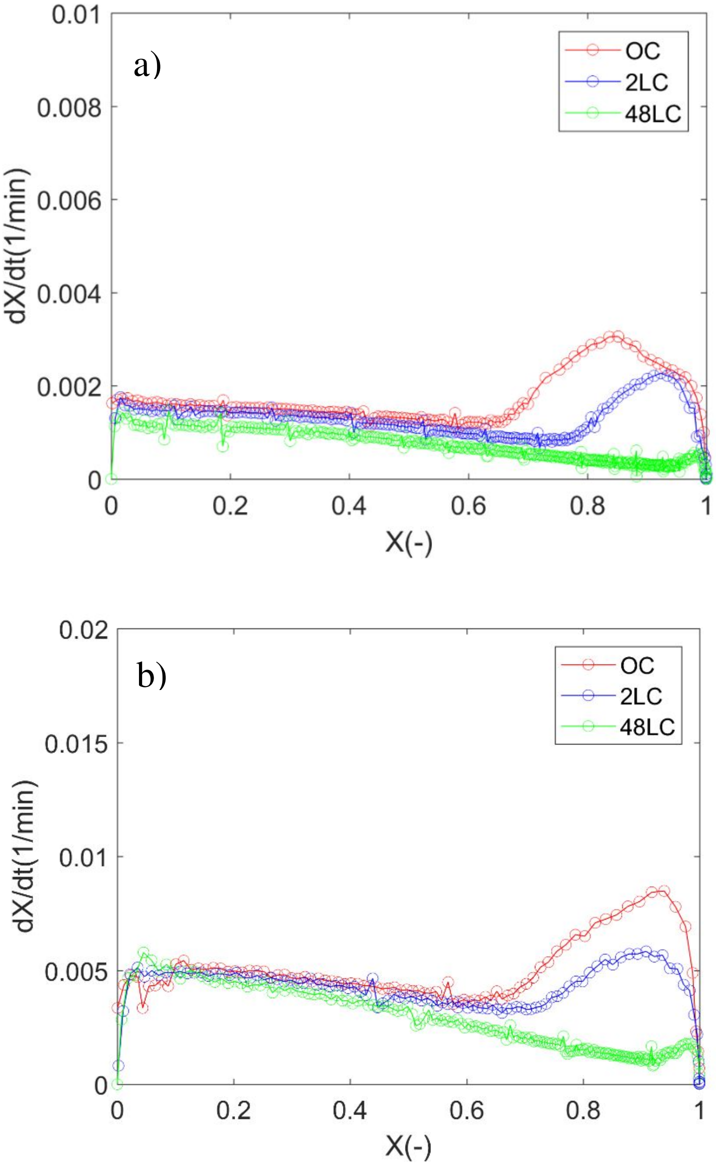

Figure S2 Experimental reactivity vs X for a) 700°C and b) 750°C for OC, 2LC and 48LC

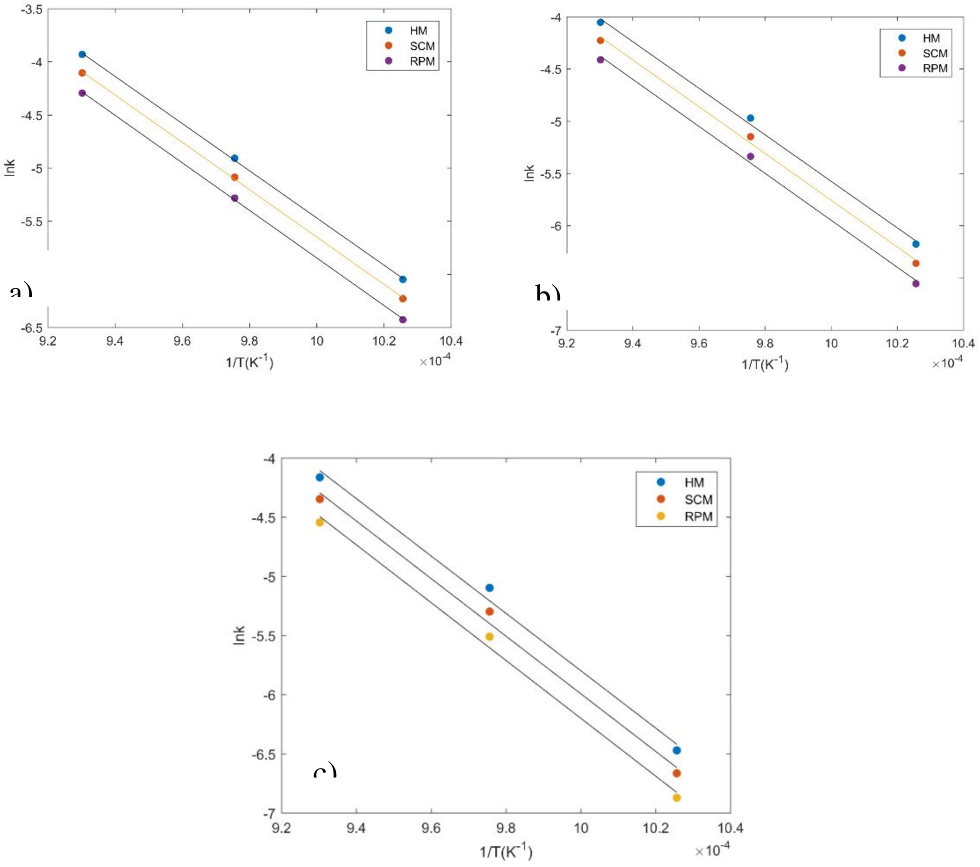

Figure S3. Arrhenius plot of three different models (a) OC (b)2LC (c) 48LC

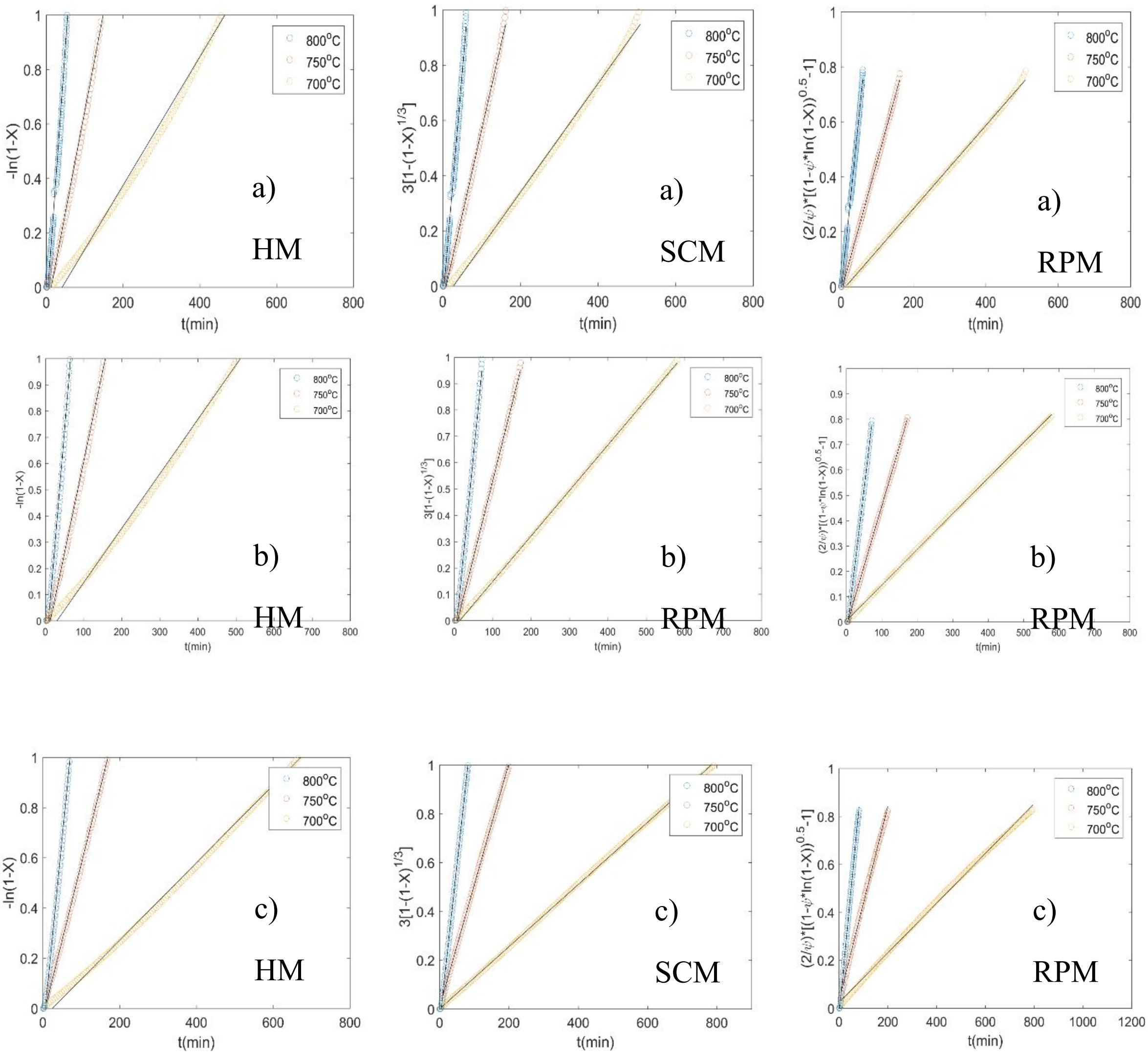

Figure S4. Linearized plot of HM, SCM and RPM models for chars at three different temperatures (a) OC, b) 2LC, c) 48LC)

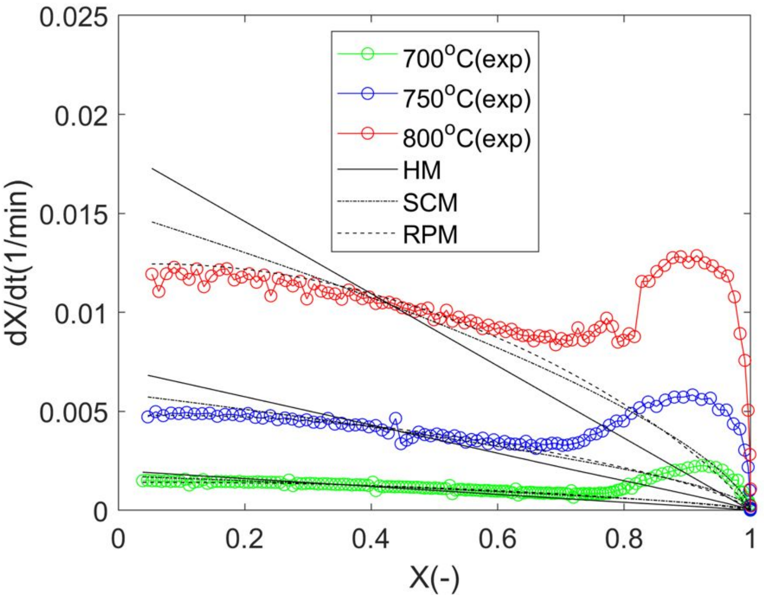

Figure S5. Comparison of the simulated and experimental data

for gasification of 2LC

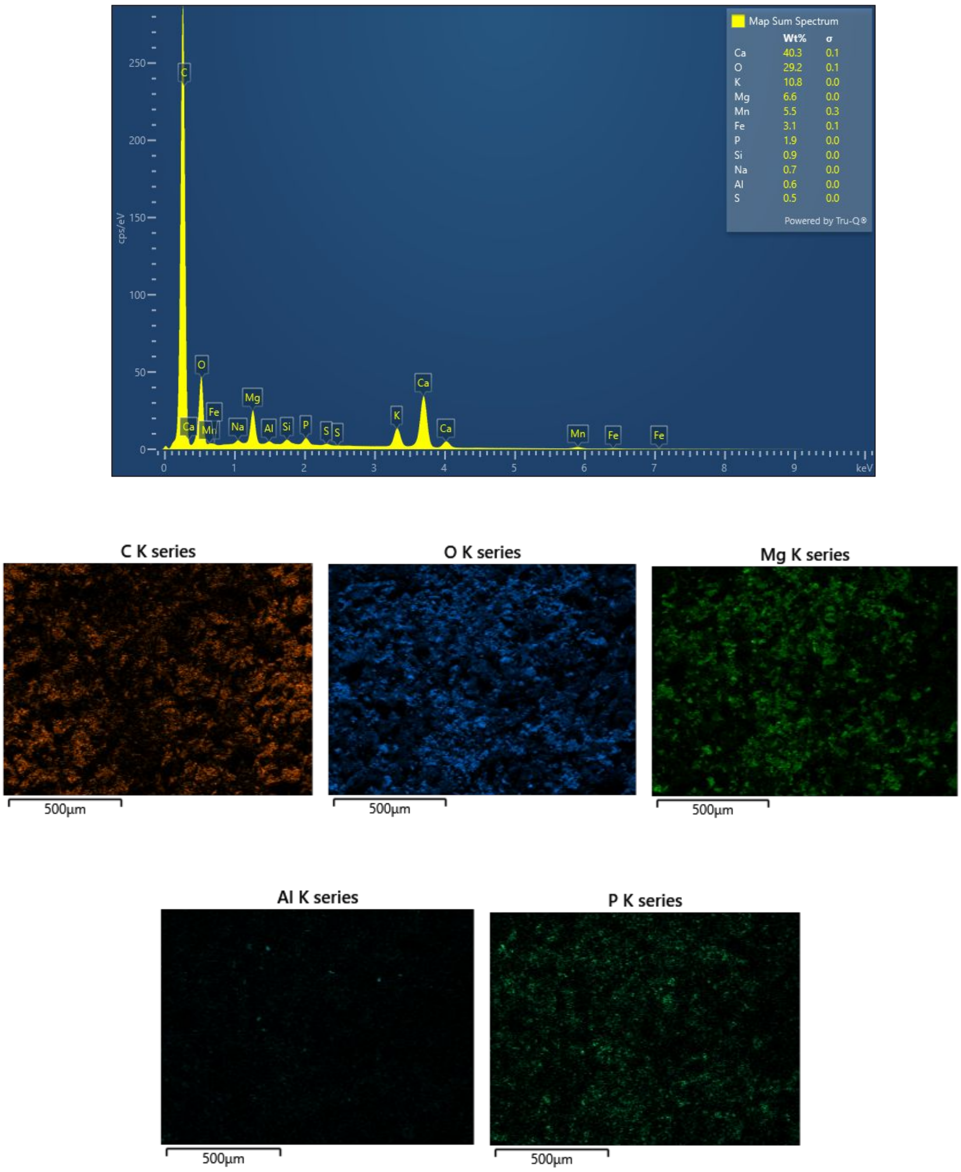

Figure S6. EDS mapping of 75% converted of OC

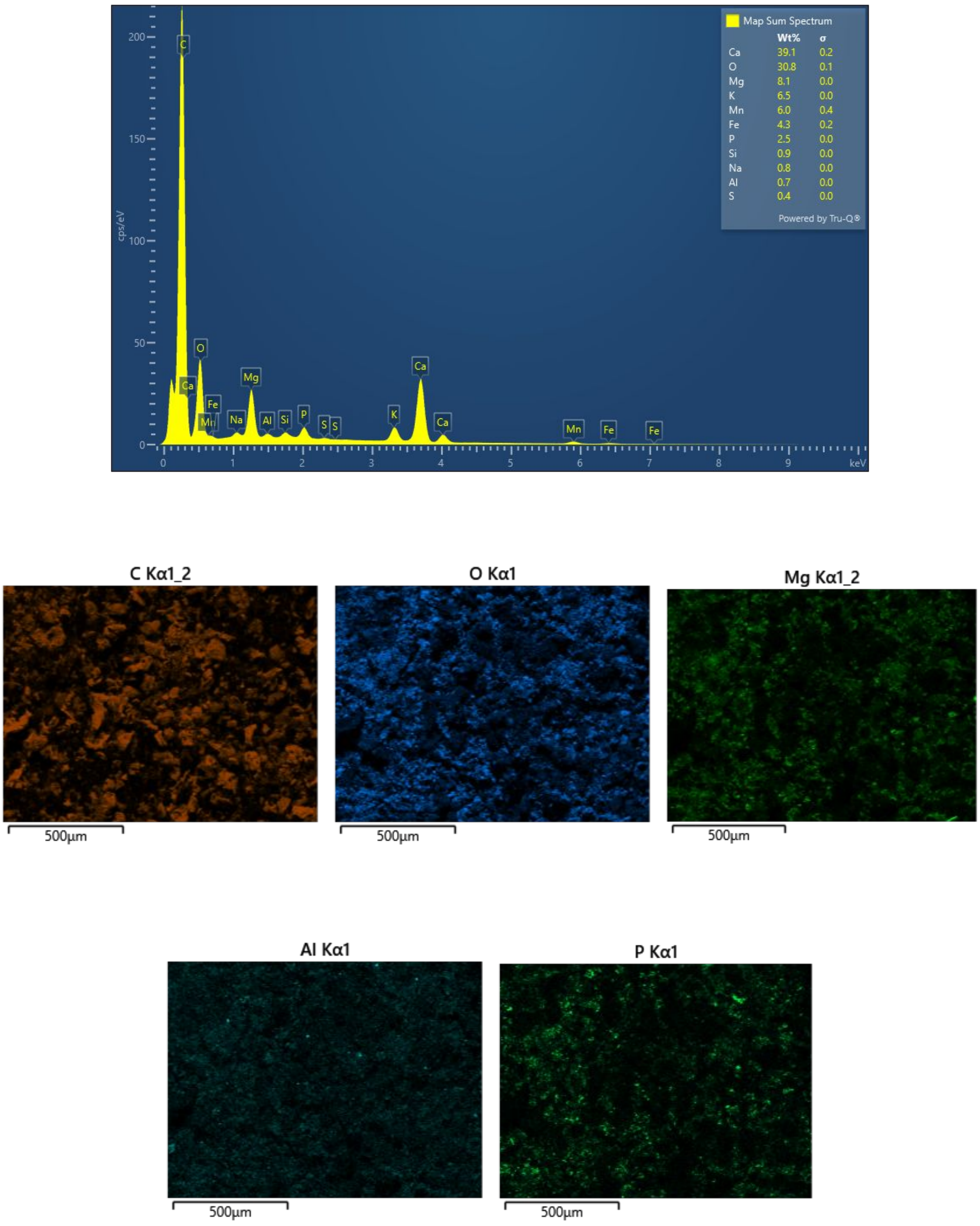

Figure S7. EDS mapping of 80% converted 2LC

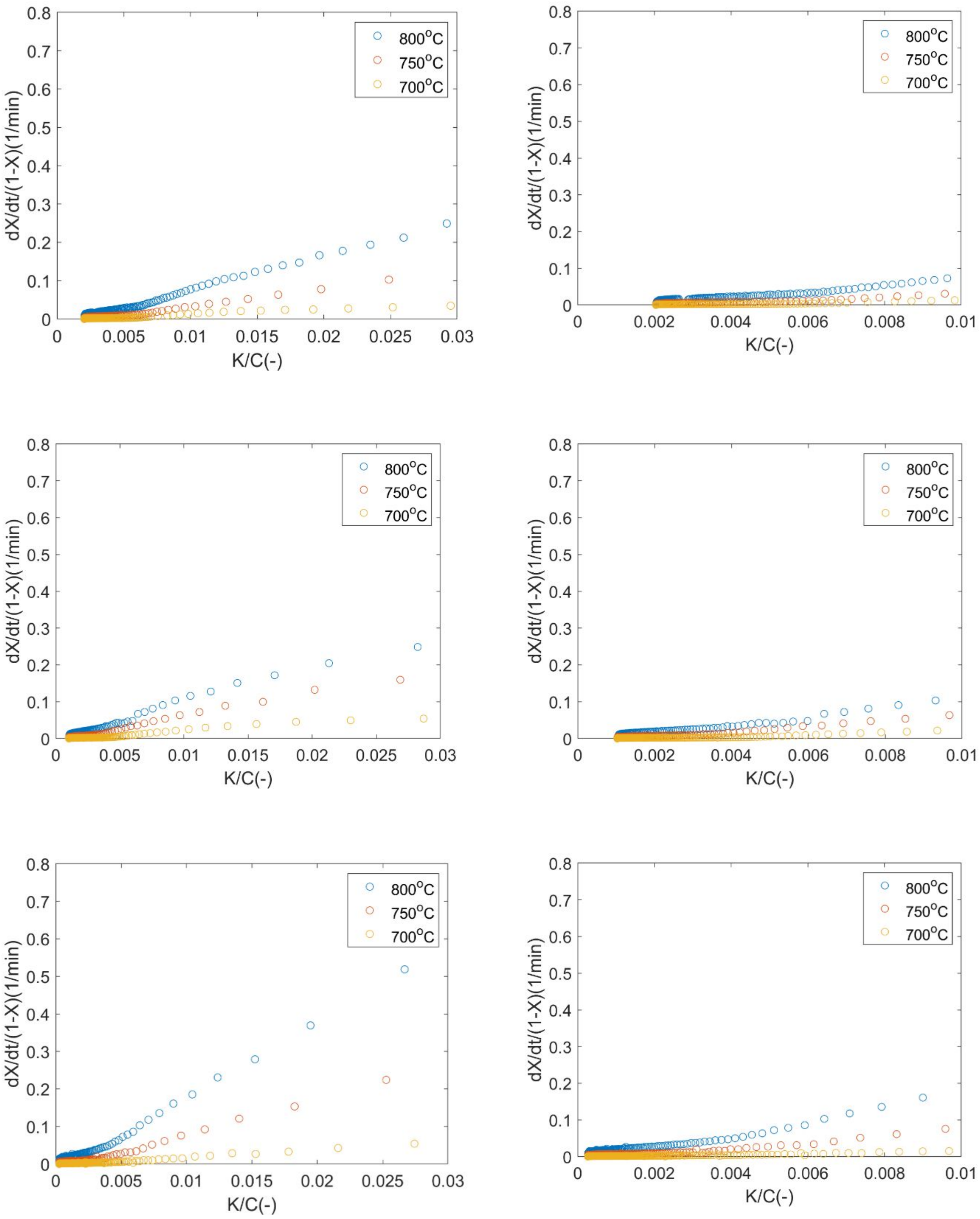

Figure S8. The K/C ratio versus instantaneous gasification rate of different char samples at different temperatures ; top row OC; middle row 2LC; bottom row 48LC.

Table S1. The kinetic parameters and regression coefficients estimated by the MRPM

Samples	T(°C)	*k* (min^{-1})	ψ(-)	c (-)	p(-)	R^2
OC	700	0.00148	2.28	1.370	9.00	0.841
	750	0.00470	2.28	1.340	9.50	0.941
	800	0.01230	2.28	1.330	8.20	0.890
2LC	700	0.00170	2.22	1.240	12.88	0.923
	750	0.00600	2.22	1.270	10.10	0.924
	800	0.01400	2.22	1.235	11.55	0.912
48LC	700	0.00170	2.17	1.056	55.77	0.927
	750	0.00395	2.17	1.06	47.96	0.95
	800	0.01050	2.17	1.06	44	0.966
